# *GJB2* Mutations Linked to Hearing Loss Exhibit Differential Trafficking and Functional Defects as Revealed in Cochlear-Relevant Cells

**DOI:** 10.3389/fcell.2020.00215

**Published:** 2020-04-02

**Authors:** Rianne Beach, Julia M. Abitbol, Brian L. Allman, Jessica L. Esseltine, Qing Shao, Dale W. Laird

**Affiliations:** ^1^Department of Anatomy and Cell Biology, Schulich School of Medicine & Dentistry, University of Western Ontario, London, ON, Canada; ^2^Division of BioMedical Sciences, Faculty of Medicine, Memorial University of Newfoundland, St. John’s, NL, Canada

**Keywords:** GJB2, hearing loss, Cx26, cochlear cells, mutants, disease

## Abstract

*GJB2* gene (that encodes Cx26) mutations are causal of hearing loss highlighting the importance of Cx26-based channel signaling amongst the supporting cells in the organ of Corti. While the majority of these *GJB2* mutations are inherited in an autosomal recessive manner, others are inherited in an autosomal dominant manner and lead to syndromic hearing loss as well as skin diseases. To assess if common or divergent mechanisms are at the root of *GJB2*-linked hearing loss, we expressed several mutants in cochlear-relevant HEI-OC1 cells derived from the developing organ of Corti. Since supporting cells of the mature mammalian organ of Corti have negligible Cx43, but HEI-OC1 cells are rich in Cx43, we first used CRISPR-Cas9 to ablate endogenous Cx43, thus establishing a connexin-deficient platform for controlled reintroduction of hearing-relevant connexins and Cx26 mutants. We found three distinct outcomes and cellular phenotypes when hearing loss-linked Cx26 mutants were expressed in cochlear-relevant cells. The dominant syndromic Cx26 mutant N54K had trafficking defects and did not fully prevent wild-type Cx26 gap junction plaque formation but surprisingly formed gap junctions when co-expressed with Cx30. In contrast, the dominant syndromic S183F mutant formed gap junctions incapable of transferring dye and, as expected, co-localized in the same gap junctions as wild-type Cx26 and Cx30, but also gained the capacity to intermix with Cx43 within gap junctions. Both recessive non-syndromic Cx26 mutants (R32H and R184P) were retained in intracellular vesicles including early endosomes and did not co-localize with Cx30. As might be predicted, none of the Cx26 mutants prevented Cx43 gap junction plaque formation in Cx43-rich HEI-OC1 cells while Cx43-ablation had little effect on the expression of reference genes linked to auditory cell differentiation. We conclude from our studies in cochlear-relevant cells that the selected Cx26 mutants likely evoke hearing loss via three unique connexin defects that are independent of Cx43 status.

## Introduction

Nearly half of all inherited sensorineural hearing loss is attributed to mutations in one of four members of the 21 connexin gene family (Chan and Chang, 2014), although *GJB2* gene (encoding Cx26) mutations linked to hereditary deafness are by far the most common (Duman and Tekin, 2012; Mammano, 2019). Connexins (Cxs) oligomerize into hexameric arrangements called connexons or hemichannels. At the cell surface, hemichannels may function as highly regulated communication conduits to the extracellular milieu but more often proceed to dock with hemichannels from a contacting cell to form gap junction channels (Laird, 2006). These channels facilitate the direct intercellular exchange of metabolites, ions, and small molecules (<1 kDa) in a process known as gap junctional intercellular communication (GJIC) (Alexander and Goldberg, 2003). The principal connexin isoforms implicated in hearing loss are Cx26 and Cx30, which are abundantly expressed in two independent gap junction networks in the cochlea: the epithelial and connective tissue networks (Kikuchi et al., 2000b; Ahmad et al., 2003; Forge et al., 2003; Liu et al., 2009). The connective tissue network exists amongst the cells of the cochlear lateral wall while the epithelial gap junction network is found amongst supporting cells that are precisely configured around the mechanosensory hair cells in the organ of Corti (Jagger and Forge, 2015). Cx26 and Cx30 also have the capacity to co-oligomerize and form heteromeric and/or heterotypic (mixed) channels within these networks enhancing the scope of GJIC and possibly hemichannel function (Yum et al., 2007; Martinez et al., 2009). Hair cells are completely devoid of connexins even though hair cell loss is a consequential outcome of connexin-based sensorineural hearing loss (Jagger and Forge, 2006; Forge et al., 2013). The exact role of connexins in supporting cell signal propagation has been extensively debated (Zhao, 2017). Hearing initiates through an influx of potassium ions into hair cells that drives their depolarization and subsequent propagation of electrical signals along the auditory nerve, ultimately relaying sensory information into the central auditory system (Wangemann, 2006). After hair cell stimulation, gap junction networks have been proposed to be important in buffering and recycling potassium ions back into the potassium-rich endolymph fluid that bathes the hair cells, and is crucial for hair cell depolarization (Kikuchi et al., 2000a; Jagger and Forge, 2015). Furthermore, gap junction networks have been demonstrated to be vital in cochlear development, homeostasis, and nutrient transfer (Zhao et al., 2006; Chang et al., 2008; Liang et al., 2012).

Approximately 135 different hearing loss mutations in the *GJB2* gene have been identified (Laird, 2008; Laird et al., 2017) that span the entire amino acid polypeptide sequence of Cx26 (Martinez et al., 2009). In an attempt to correlate genotype changes to phenotype outcomes, some of these mutants have been expressed and examined in tumor cells and other cells unrelated to hearing. Based on these studies, connexin mutants can be categorized as exhibiting either loss-of-function or gain-of-function properties (Kelly et al., 2014; Verselis, 2019). Loss-of-function mutants can result in defective trafficking of the Cx26 mutant through the endoplasmic reticulum (ER) and Golgi apparatus, misfolding and aberrant oligomerization, and non-functional hemichannels and/or gap junction formation (Laird, 2008; Kelly et al., 2015). In contrast, abnormal oligomerization of a Cx26 mutant with other connexin isoforms, formation of leaky hemichannels, formation of hyperactive hemichannels and/or gap junctions are all characteristics of gain-of-function mutants (Press et al., 2017; Srinivas et al., 2018). Loss-of-function Cx26 mutants typically produce hearing loss as the pathological outcome and are characterized as non-syndromic mutations, where hearing loss is the only phenotype (Kenneson et al., 2002). Gain-of-function Cx26 mutants frequently result in syndromic disease, where hearing loss is also accompanied with other co-morbidities, as these mutants often induce a skin disorder (Srinivas et al., 2018). Evidence suggests that gain-of-function Cx26 mutants induce skin disorders because of their inhibitory trans-dominant effects on other connexin isoforms expressed in the epidermis (Press et al., 2017). In all cases, Cx26 mutants drive moderate to profound hearing loss raising questions as to whether this is rooted in how the Cx26 mutants are trafficked, assembled, and functionally dysregulated (D’Andrea et al., 2002; Snoeckx et al., 2005; Xiao et al., 2011). Because of the diversity and extent of hearing loss that occurs when Cx26 mutants are expressed in the organ of Corti, the mechanisms of hearing loss need to be investigated in a tissue-relevant setting.

Hair cells and supporting cells develop from common progenitor cells within the prosensory domain of the developing cochlea. At an early stage of development, specification of cell fate depends on the crucial coordination and timing of gene expression (Basch et al., 2016). The expression of Cx26 within the epithelial gap junction network begins to occur around embryonic day 16 in mice (Frenz and Van De Water, 2000) and continues for approximately two weeks after birth as mouse hearing matures. As revealed in mouse studies, improper cochlear development is a pathological outcome of Cx26 mutant expression or Cx26 ablation as noted by the deformation of hair cells and disrupted formation of the tunnel of Corti, which is formed by supporting cells (Wang et al., 2009; Mese et al., 2011; Schutz et al., 2011; Inoshita et al., 2014; Anzai et al., 2015; Lee et al., 2015; Zhu et al., 2015; Chen et al., 2018b). A few rare mutations in *GJC3* (Cx30.2/Cx29) and *GJB3* (Cx31) have also been linked to hearing loss but it is unclear what role these connexins play and even where these connexins are localized in the auditory tract (Wingard and Zhao, 2015). Cx43 is expressed early on in cochlear development, however Cx43 expression is negligible in the mature organ of Corti (Cohen-Salmon et al., 2004) although mice expressing a loss-of-function G60S Cx43 mutant were found to have severe hearing loss (Abitbol et al., 2018).

Since *GJB2* is the primary connexin gene linked to sensorineural hearing loss (Johnson et al., 2017) and its mechanism of action in the cochlea remains uncertain, it is the connexin of focus in the present study. Cx26 has been shown to facilitate the passage of miRNAs necessary for coordinated development and differentiation of the organ of Corti (Zhao, 2017). Thus, Cx26 status may impact the expression of key factors necessary for proper organ of Corti formation. These factors include the Sox2 transcription factor, which is necessary for the designation of the prosensory domain containing progenitor cells (Atkinson et al., 2018). Increased expression of the transcription factor Atoh1 is essential for the initiation of hair cell differentiation (Chonko et al., 2013). Many other proteins are exclusively expressed in mature hair cells such as the motor protein prestin, unconventional myosin proteins, and calcium binding proteins (Hasson et al., 1995; Zheng et al., 2000; Keller et al., 2014). Nevertheless, the mechanisms underpinning how Cx26 mutations and aberrant Cx26 channel function influences gene expression as well as differentiation and maintenance of hair cells within the organ of Corti remains unclear.

In order to examine Cx26 mutants in a more hearing-relevant cellular context, we employed HEI-OC1 cells derived from the progenitor region of P7 mouse cochlear explants, associated with the epithelium of the organ of Corti. These cochlear-relevant cells have been shown to differentiate into both supporting cells and hair cell-like cells that express hair cell specific genes (Kalinec et al., 2003; Kalinec G. M. et al., 2016; So et al., 2005; Park et al., 2016). HEI-OC1 cells have been utilized as a model to study cell fate and differentiation, and the onset of hearing loss that occurs after therapeutic drug usage (Youn et al., 2015; Kalinec G. M. et al., 2016; Kim et al., 2016; Pang et al., 2018; Choi et al., 2019; Lim et al., 2019). Surprisingly, HEI-OC1 cells lack the protein expression of Cx26 and Cx30 found in the organ of Corti, but abundantly express Cx43. In the current study, we selected four different missense *GJB2* mutations, which result in either syndromic or non-syndromic hearing loss, in order to compare and contrast their cellular localization and function in auditory cells before and after Cx43 ablation. Collectively, we identified that the selected Cx26 mutants acquired three cellular phenotypes that underpin how they cause either syndromic or non-syndromic hearing loss.

## Materials and Methods

### Cell Culture and Reagents

House Ear Institute-Organ of Corti 1 (HEI-OC1) cells were generously provided by Dr. Kalinec (House Ear Institute, Los Angeles, CA) (Kalinec et al., 2003; Kalinec G. et al., 2016; Kalinec G. M. et al., 2016; Kelly et al., 2019). HEI-OC1 cells were grown as we recently described (Abitbol et al., 2020). To induce hair cell-like cell differentiation, HEI-OC1 cells that were ∼80% confluent were transferred into non-permissive conditions (39°C and 5% CO_2_) for ten days and regular media was replenished every other day to remove dead cells.

### Cell Engineering

The *Gja1* gene encoding Cx43 was ablated from mouse HEI-OC1 cells using a CRISPR-Cas9 strategy as we described (Abitbol et al., 2020). These Cx43-null cells are referred to as Cx43 knockout (KO) cells. Constructs encoding wild type (WT) Cx26, Cx30, and Cx26 mutants (N54K, S183F, R32H, and R184P) were sub-cloned into moxGFP vectors (Addgene). Sequences were verified by NorClone Biotech Laboratories. Cx26-RFP and Cx30-RFP were generated as previously described (Berger et al., 2014). HEI-OC1 cells at ∼60% confluency in six well dishes were transiently transfected with 1 μg of the desired cDNA construct using Mirus TransIT-LT1 Transfection Reagent (Cat# MIR2304, Mirus Bio). Co-transfection of Cx26-RFP and Cx30-RFP with Cx26 mutant constructs were done at a 1:1 ratio consisting of 0.75 μg of each cDNA vector to approximate equal protein expression. Cells were then fixed ∼30 h after a successful transfection. In some cases where cells were prepared for imaging, HEI-OC1 cells that lacked Cx43, were grown on 35 mm glass bottom dishes coated with sterile filtered type I rat tail collagen (Cat# 354236, Corning Life Sciences) diluted in 0.02 M acetic acid for one hour. Once cells were ∼60% confluent they were transfected with 1 μg of either Cx26-GFP or S183F-GFP cDNA constructs using Mirus TransIT-LT1 Transfection Reagent. For all experiments involving Cx43 KO cells, two independent CRISPR clones were used and pooled together for analysis.

### Western Blotting and Immunofluorescence

Western blotting for Cx43, Cx30, Cx26, and GAPDH was performed on HEI-OC1 cell lysates using immunoblotting procedures as we described (Abitbol et al., 2020). Primary antibodies included: mouse anti-GAPDH (1:5000, Cat# MAB374, EMD Millipore), rabbit anti-GAPDH (1:5000, Cat# G9545, Sigma), rabbit anti-Cx43 (1:5000, Cat# C6219, Sigma), mouse anti-Cx26 (1:1000, Cat# 138100, Life Technologies), and rabbit anti-Cx30 (1:1000, Cat# 712200, Life Technologies). For immunofluorescence, HEI-OC1 cells grown on glass coverslips were fixed with 4% paraformaldehyde for 10 minutes prior to being washed with phosphate-buffered saline (PBS). HEI-OC1 cells treated with 0.1% Triton X-100 + 3% bovine serum albumin (BSA) for one hour were incubated with primary antibodies diluted in 0.1% Triton X-100 + 3% BSA overnight at 4°C. Primary antibodies included: rabbit anti-Cx43 (1:750, Cat# C6219, Sigma), mouse anti-Cx26 (1:200, Cat# 138100, Life Technologies), rabbit anti-Cx30 (1:200, Cat# 712200, Life Technologies), mouse anti-GM130 (1:500, Cat# 610822, BD Biosciences), rabbit anti-EEA1 (1:500, Cat# ab2900, Abcam), rabbit anti-prestin (1:200, Cat# AV447176, Sigma), and mouse anti-Sox2 (1:50, Cat# sc-365823, Santa Cruz). Coverslips were washed with PBS and incubated with secondary antibodies diluted in 0.1% Triton X-100 + 3% BSA for one hour at room temperature. Secondary antibodies included: goat anti-mouse 633 (Cat# A21052, Invitrogen), goat anti-rabbit 568 (Cat# A11036, Invitrogen), and goat anti-mouse 555 (Cat# A21422, Invitrogen). Cells were stained with Hoechst (1:1000 diluted in distilled H_2_O, Cat# H3570, Molecular Probes) for 10 minutes to visualize the nuclei and coverslips were mounted using Airvol. C57BL/6 mouse cochleae were dissected and used in a cell lysate or cryosectioned, and immunolabeled as previously described (Kelly et al., 2019). Mouse usage for this purpose was approved by the Animal Care Committee at the University of Western Ontario. Cell images were captured using a Zeiss LSM800 confocal microscope equipped with airyscan and a 63x oil immersion objective. Representative images of wild-type connexins and Cx26 mutants were selected from a minimum of three independent transfections involving four coverslips per treatment and after interrogation of dozens of transfected cells per coverslip.

### Scrape Loading Dye Transfer

Wild type and Cx43-ablated HEI-OC1 cells were seeded onto culture dishes coated with sterile filtered type I rat tail collagen diluted in 0.02 M acetic acid. Once cells were ∼80% confluent they were washed twice with Hank’s balanced salt solution (HBSS) and a scrape line was made in the presence of the gap junction permeable molecule, neurobiotin (2 mg/ml, Cat# SP-1120, Vector), and the gap junction impermeable molecule, dextran rhodamine (0.5 mg/ml, Cat# D1824, Invitrogen). After five minutes at 33°C and 10% CO_2_, the cells were washed with HBSS and fixed for 10 min in 4% paraformaldehyde. Cells were permeabilized in 0.1% Triton X-100 for 30 min, before incubating for one hour in the presence of Alexa Fluor 488- conjugated streptavidin (1:1000, Cat# S11223, Invitrogen) to label the trapped neurobiotin. Samples were imaged using a Zeiss LSM 800 confocal microscope equipped with a 10x objective. In three independent experiments, a minimum of six images were taken per experiment and four measurements per image were collected for a total of at least 72 individual measurements. ImageJ was used to measure the distance of neurobiotin spread (μm) beyond the first row of damaged cells along the scrape line and an unpaired *t*-test was performed.

### Fluorescence Recovery After Photobleaching (FRAP)

Cell cultures expressing Cx26-GFP or S183F-GFP were incubated in HBSS containing 2 mM calcein-AM for five minutes at room temperature. Cell cultures were washed with HBSS and replenished with warm media at 33^o^C prior to imaging selected regions where adjacent cells expressed fluorescent protein-tagged Cx26 or S183F. A region of interest (ROI) was photobleached to ∼30% of initial fluorescence intensity. FRAP images were captured every 10 seconds for five minutes and dye recovery within the ROI was determined using the Time Series Analyzer V3 plugin on ImageJ. For each ROI, fluorescence recovery was measured using Recovery (%) = (F_t_ − F_0_/F_b_) × 100 (F_t_ fluorescence at each time point after photobleaching, F_0_: fluorescence at 0 s after photobleaching, F_b_: fluorescence before photobleaching) (Simek et al., 2009). Fluorescence recovery was plotted as an average of three replicates each consisting of a minimum of four pairs of connexin or mutant expressing cells. The mean area under the curve was then calculated and compared using an unpaired *t*-test. Negative controls consisted of FRAP imaging of Cx43-ablated HEI-OC1 cells.

### Quantitative Real Time Polymerase Chain Reaction

Total RNA was collected from permissive and non-permissive HEI-OC1 cells after differentiation using RNeasy Mini Protocol for Isolation of Total RNA from Animal Cells (Cat# 74106, Qiagen) and was converted to cDNA using the High Capacity cDNA Reverse Transcription Kit (Cat# 4368814, Applied Biosystems). qRT-PCR was conducted using PowerUp SYBR Green Master Mix (Cat# A25742, Life Technologies) and the cycle conditions for each primer consisted of: 50°C for 2 min, 95°C for 2 min, 95°C for 5 s, and 60°C for 15 s for 40 cycles, followed by a melt curve. The following primers were used: 18s rRNA, the house keeping gene (forward, 5′-GTAACCCGTTGAACCCCATT; reverse, 5′-CCATCCAATCGGTAGTAGCG), Atoh1 (forward, 5′- GAGTG GGCTGAGGTAAAAGAGT; reverse, 5′- GGTCGGTGCTA TCCAGGAG), calsequestrin (forward, 5′- CGAGACTTGGG AGGATGACC; reverse, 5′- TCGGGGTTCTCAGTGTTGTC), myosin VIIa (forward, 5′- TGGTACACTTGACACTGAAG; reverse, 5′- CCATCGTTCAGCCTCTTGGT), and nestin (forward, 5′- GCTGGAACAGAGATTGGAAGG; reverse, 5′- CCAGGATCTGAGCGATCTGAC). mRNA levels were normalized to 18S rRNA levels and measured using the 2^–ΔΔ*C*^*^T^* method.

### Statistical Analysis

A two-way ANOVA and a Tukey’s *post hoc* test was used to determine statistical significance between mRNA expression of permissive and non-permissive WT and Cx43-ablated cells. An unpaired *t*-test was used to compare mRNA expression before and after differentiation.

All statistical analysis was conducted using Graph Pad Prism 6 and results were indicated as statistically significant when *P* < 0.05. Outliers were removed using the ROUT method with Q set to 1%. Results are presented as mean ± SEM unless stated otherwise.

## Results

### HEI-OC1 Cells Express Cx43 but Become GJIC-Deficient Upon Its Ablation

**FIGURE 1:**
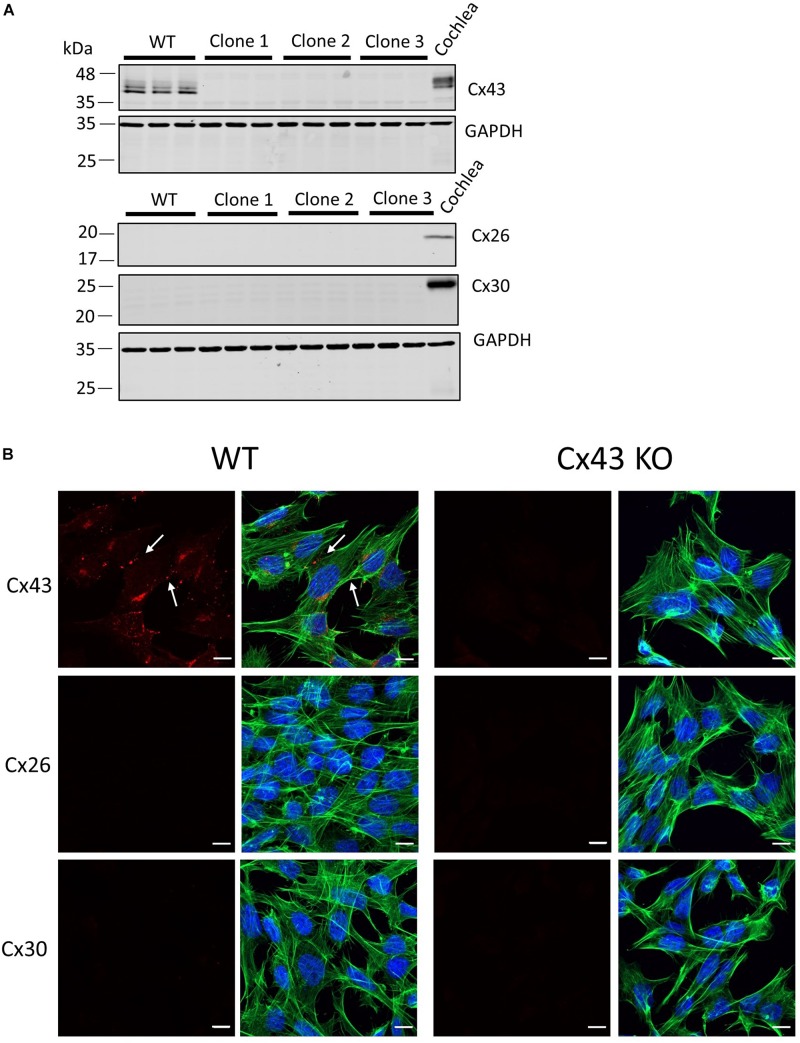
Characterization of connexin expression and Cx43 ablation in HEI-OC1 cells. **(A)** Western blots for Cx43, Cx26, and Cx30 protein in wild type (WT) and three independent clones of CRISPR-Cas9 Cx43-knockout (Cx43-KO) HEI-OC1 cells where adult mouse cochlear lysate was used as a positive control. Molecular weight standards are denoted in kDa. **(B)** Immunolabeling revealed Cx43 gap junctions only in WT cells denoted by white arrows. Note Cx26 and Cx30 were not detected in WT or Cx43 KO cells **(A,B)**. Red = Cx43, Cx26, and Cx30, green = phalloidin staining of actin filaments, blue = Hoechst stained nuclei. Bars = 10 μm.

Previous studies have used cells unrelated to the organ of Corti to characterize the etiology of several hearing loss-linked Cx26 mutants. Here we employed HEI-OC1 cells (Kalinec et al., 2003; Kalinec G. M. et al., 2016) that were derived from the progenitor epithelium of the organ of Corti from P7 mice as a tissue-relevant cell model to assess several Cx26 mutants that have been associated with non-syndromic and syndromic hearing loss. Since western blotting and immunofluorescence revealed that these cells were rich in Cx43 (Figure 1A), we used CRISPR-Cas9 to genetically ablate the *Gja1* gene and all subsequent Cx43 expression from these cells (Figures 1A,B). Cx43 is not an endogenous connexin typically expressed in the mature organ of Corti (Forge et al., 2003), but is often upregulated in cultured cells. Not surprisingly since the expression of most connexin gene isoforms are silenced in cultured cells, neither Cx26 or Cx30 were expressed in WT cells or found in cells lacking Cx43 (Figures 1A,B).

**FIGURE 2:**
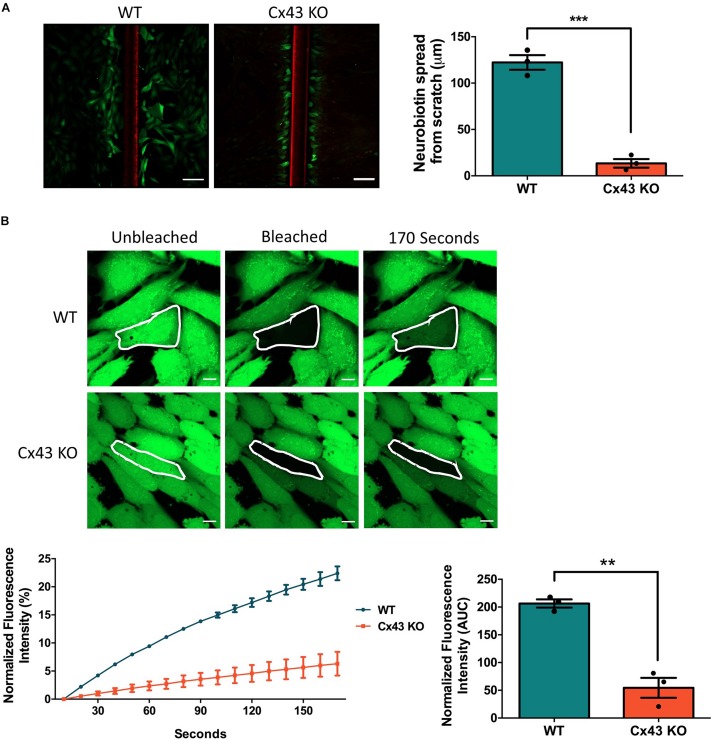
Ablation of Cx43 greatly reduces dye transfer. **(A)** Representative micrographs of a scrape loading dye transfer assay performed on WT and Cx43 KO HEI-OC1 cells. Cells were scraped and incubated with the gap junction permeable tracer neurobiotin (green) and impermeable dextran rhodamine dye (red) that typically gets washed out during the sample preparation. Cx43 KO cells exhibited significantly less neurobiotin transfer from the first row of damaged cells than WT cells. **(B)** Representative micrographs of WT and Cx43 KO cells loaded with calcein-AM and subjected to fluorescence recovery after photobleaching of a selected cell (outlined in white). Dye recovery and area under the curve (AUC) was measured over 170 s. Cx43 KO cells had significantly less dye recovery after photobleaching compared to WT cells. Data represent mean ± SEM from three independent experiments and were analyzed using an unpaired *t*-test. ***p < 0.001, **p < 0.01. Bars in **(A)** = 100 μm and **(B)** = 10 μm.

To investigate the consequence of Cx43 ablation on GJIC, confluent cultures of WT HEI-OC1 cells and cells lacking Cx43 were scraped and incubated with a gap junction permeable positively charged small molecule, neurobiotin (287 Da). Neurobiotin was found to spread beyond the first row of damaged cells to an average distance of 122.3 μm in WT cells, but in Cx43 KO cells neurobiotin travelled only an average distance of 13.4 μm (Figure 2A). FRAP was also completed to further determine the level of GJIC in HEI-OC1 cells using a negatively charged gap junction permeable dye calcein-AM (623 Da). FRAP revealed that the Cx43-rich WT cells were significantly more capable of passing calcein through gap junctions than Cx43 KO cells (Figure 2B). These findings demonstrate that WT HEI-OC1 cells have abundant gap junction function, while the lack of Cx43 greatly reduced GJIC. Thus, Cx43 KO cells were utilized as GJIC-deficient in subsequent experiments, where Cx26 mutants could be expressed in a connexin-deficient cochlear-relevant cell system.

### Differential Distribution of Cx26 Mutants in Cx43 KO Cells

**FIGURE 3:**
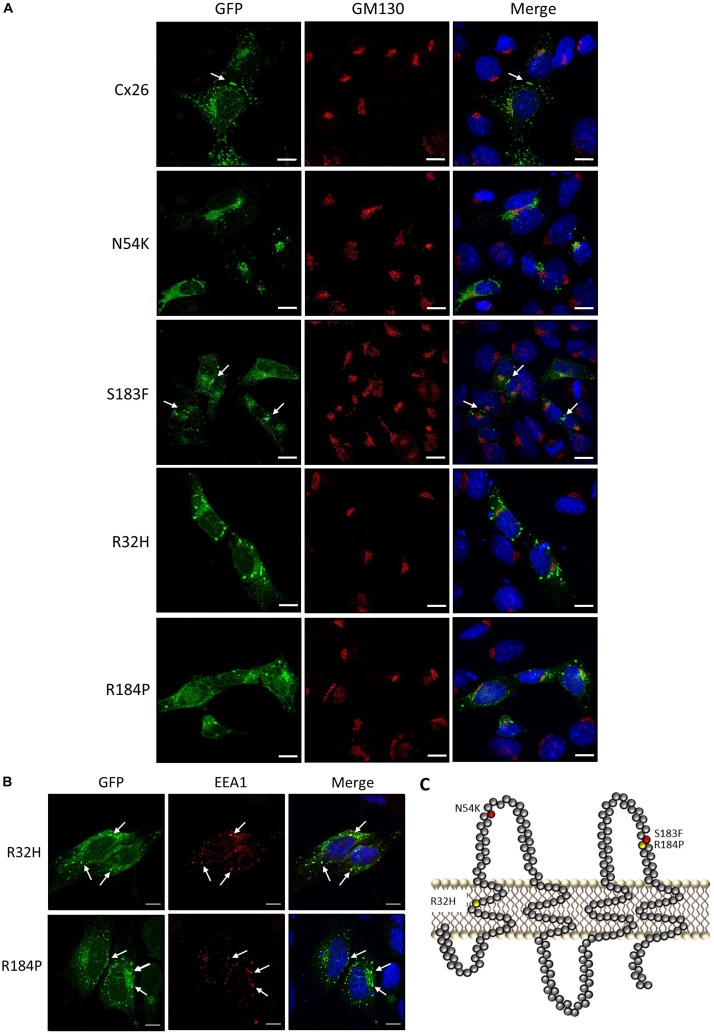
Intracellular localization of syndromic and non-syndromic hearing loss-linked Cx26 mutants **(A)** Representative micrographs of Cx26-GFP and various GFP-tagged hearing loss-linked Cx26 mutants transiently expressed in Cx43 KO cells. Cells were co-immunolabeled with GM130 (red) to visualize the location of the Golgi apparatus and Hoechst (blue) to visualize the nuclei. The N54K, R32H, and R184P mutants failed to form gap junctions. Arrows denote gap junction plaques. **(B)** A sub-population of the R32H and R184P mutants co-localized with EEA1, an early endosomal marker, denoted by arrows. **(C)** Topological model of Cx26 depicting the approximate locations of the syndromic (red) and non-syndromic (yellow) mutations. Bars = 10 μm.

To assess the localization of various hearing loss-linked Cx26 mutants, WT Cx26 and N54K, S183F, R32H, and R184P Cx26 mutants were expressed in Cx43 KO cells. Cx26 successfully trafficked to the plasma membrane and gap junction plaques were readily found (Figure 3A). In comparison to WT Cx26, the syndromic N54K mutant was retained within an intracellular compartment, but did not colocalize well with the GM130 resident protein of the Golgi apparatus (Figure 3A). In contrast, the syndromic S183F mutant was able to traffic to the plasma membrane and form gap junction plaques (Figure 3A), although some intracellular reservoirs of the mutant were found. Both recessive non-syndromic R32H and R184P mutants did not form clearly identifiable gap junction plaques but appeared to be partially localized within intracellular vesicles (Figure 3A). Some of these intracellular vesicles containing R32H and R184P immunolabeled for EEA1, an early endosomal marker (Figure 3B). Overall, the S183F mutation found within the 2nd extracellular loop (Figure 3C), was the mutant most capable of forming gap junction plaques in Cx43 KO cells.

### The S183F Mutant Forms Gap Junction Channels Incapable of Dye Transfer

**FIGURE 4:**
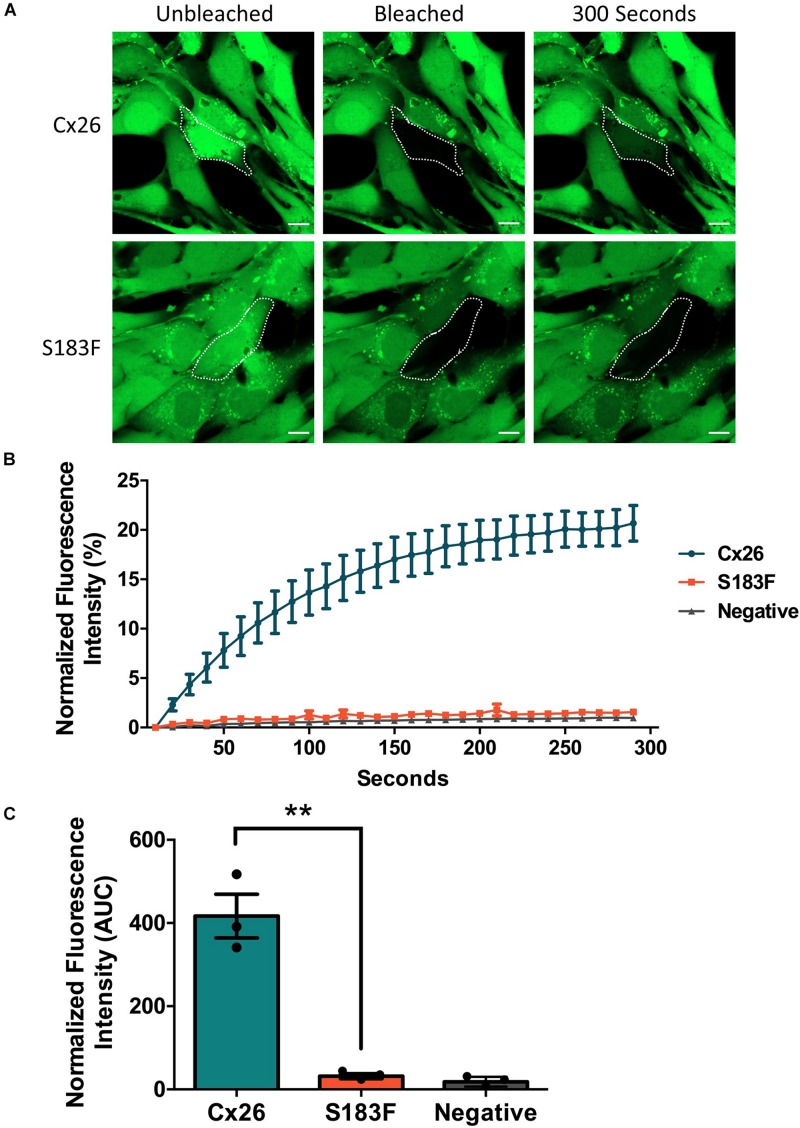
The S183F mutant does not form dye-permeable gap junction channels. **(A)** Negative (Cx43 KO cells) or Cx43 KO cells expressing Cx26-GFP or S183F-GFP were loaded with gap junction permeable dye calcein-AM (green) and subjected to FRAP. **(B)** A selected cell within a pair or cluster of Cx26 or S183F mutant expressing cells was photobleached and dye recovery over 300 s was measured. **(C)** Area under the curve (AUC) of fluorescence recovery was measured. Cx43 KO cells and cells expressing the S183F mutant had negligible dye recovery while WT Cx26 expressing cells exhibited significant dye recovery. Data represents mean ± SEM of three independent experiments and were analyzed using an unpaired *t*-test, **p < 0.01. Bars = 10 μm.

FRAP was conducted using calcein-AM dye to analyze whether the S183F mutant formed gap junction channels capable of dye transfer. Cx43 KO cell pairs or cell clusters expressing Cx26 or the S183F mutant were subjected to FRAP as a surrogate to measure GJIC. S183F-GFP expressing cells, similar to Cx43 KO cells, exhibited essentially no fluorescence dye recovery after photobleaching compared to Cx26-GFP expressing cells, quantified by measuring the area under the curve (Figures 4A–C).

### Cx30, but Not Cx26, Can Rescue the Assembly of the N54K Mutant Into Gap Junctions

**FIGURE 5:**
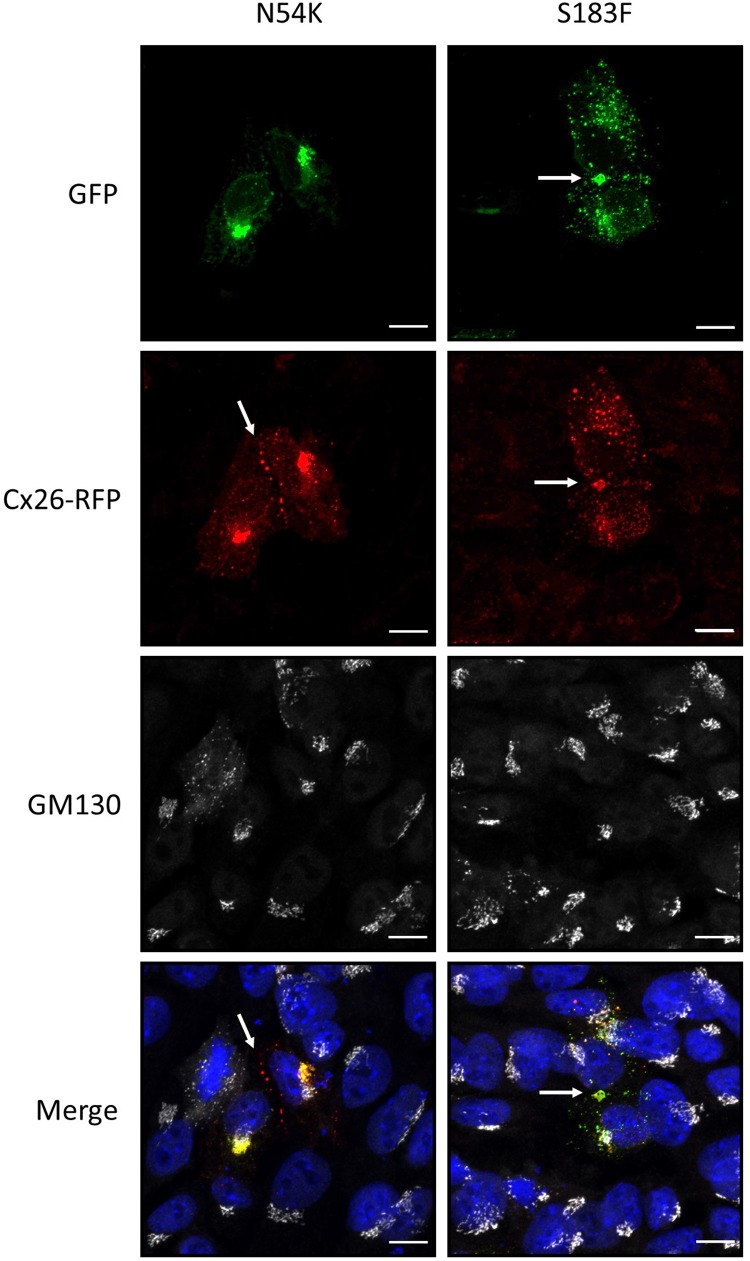
The expression of Cx26-RFP did not alter the distribution of the N54K or S183F mutant. Representative micrographs of Cx43 KO HEI-OC1 cells co-expressing Cx26-RFP and N54K-GFP or S183F-GFP. Cells were immunolabeled with GM130 (white) to visualize the location of the Golgi apparatus and counterstained with Hoechst (blue) to demarcate the nuclei. Bars = 10 μm.

Since autosomal dominant inherited Cx26 mutants are co-expressed with WT Cx26, we examined whether GFP-tagged N54K and S183F mutants might alter the intracellular localization of RFP-tagged WT Cx26. While both the S183F mutant and WT Cx26 could be found within the same gap junctions at the cell surface and within intracellular structures, the N54K mutant colocalized with WT Cx26 within intracellular stores but not typically with Cx26-RFP gap junction plaques (Figure 5). This suggests that WT Cx26 could not intermix with the N54K mutant and rescue its assembly into gap junction plaques but rather the N54K mutant impeded the trafficking of Cx26.

**FIGURE 6:**
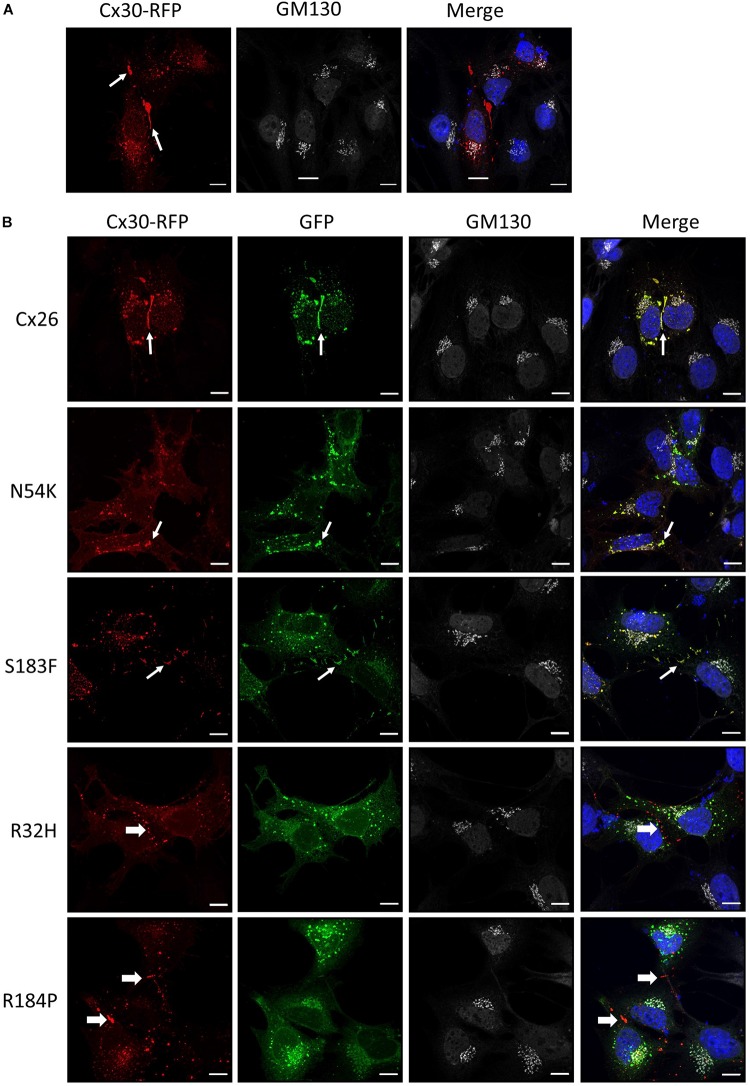
Cx30 expression partially rescued the aberrant localization of the N54K mutant. **(A)** Cx30-RFP assembled into gap junctions (arrows) when expressed in Cx43 KO cells that were immunostained for GM130 (white) and counterstained with Hoechst (blue) to demarcate the nuclei. **(B)** Cx30 and co-expressed Cx26 or the S183F and N54K mutants localized within the same gap junction plaques, denoted by the thin white arrows. Both recessive mutants, R32H and R184P, remained intracellularly and failed to co-localize with Cx30 (thick arrows). Bars = 10 μm.

Cx30 is also highly expressed in the organ of Corti and co-oligomerizes with Cx26 to form mixed gap junction plaques *in vivo* (Ahmad et al., 2003). As might be expected, we found that Cx30-RFP could form gap junctions in Cx43 KO cells (Figure 6A). We next tested if this Cx30 tissue-relevant connexin could interact with the Cx26 N54K mutant and potentially rescue its assembly into cell surface gap junctions. As also observed for the S183F mutant, the N54K mutant extensively co-localized with Cx30-RFP including within many gap junctions at sites of cell-cell apposition. However, the autosomal recessive inherited R32H and R184P mutants showed very little co-localization with Cx30 (Figure 6B). These results suggest that both the syndromic mutants (N54K and S183F) have transdominant properties and intermix with Cx30 while the non-syndromic mutants (R32H and R184P) do not.

### The S183F Mutant Intermixes With Endogenous Cx43 Gap Junctions

**FIGURE 7:**
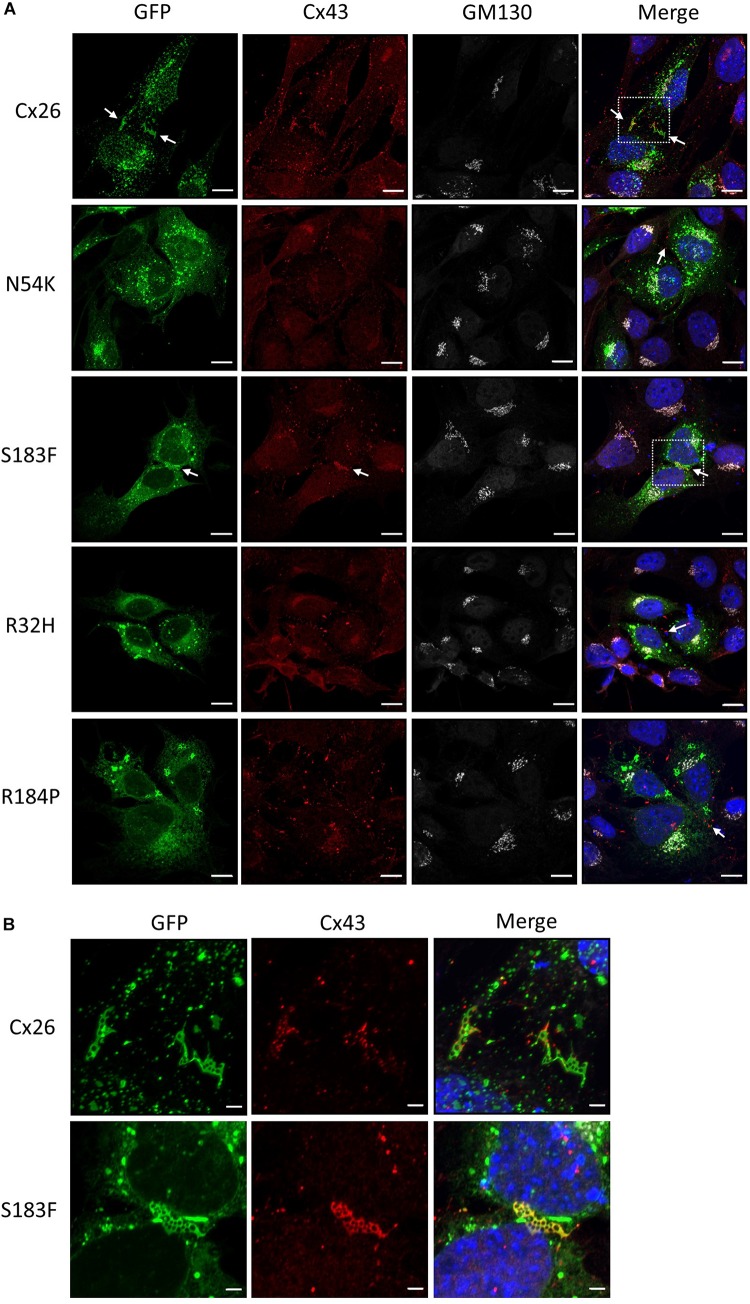
The S183F mutant intermixes with endogenous Cx43 within the same gap junctions. **(A)** Representative micrographs of wild type HEI-OC1 cells expressing Cx26-GFP or Cx26 mutants, and co-immunolabeled for Cx43 (red) and GM130 (white) prior to counterstaining with Hoechst (blue). The N54K, R32H, and R184P mutants typically localize to sites that do not contain Cx43 while WT Cx26 and the S183F mutant appear to closely associate with the position of Cx43 (arrows). **(B)** High magnification of dashed boxed image areas in **(A)** revealed that WT Cx26 segregates to gap junction domains that are part of a Cx43 gap junction (note the separation of red and green signals). However, *en face* imaging of S183F expressing cells revealed that Cx43 and the S183F mutant fully intermixed as revealed by the yellow color. Bars = 10 μm.

The expression of Cx43 in the mature organ of Corti appears negligible, however Cx43 is temporally expressed during development of the human and mouse inner ear (Cohen-Salmon et al., 2004; Locher et al., 2015). Hearing loss-linked Cx26 mutants may therefore be co-expressed with Cx43 during the development of the inner ear. To assess whether Cx26 mutants have gain-of-function properties and intermix with Cx43 within the same gap junctions, the Cx26 mutants were expressed in WT HEI-OC1 cells that contain endogenous Cx43. Previous studies have shown that Cx26 and Cx43 do not intermix to form heteromeric gap junctions under physiological conditions, but are able to partition to separate domains within the same gap junction plaques (Falk, 2000; Gemel et al., 2004). Both Cx26 and Cx43 formed gap junction plaques in HEI-OC1 cells, however, they appeared to segregate within different regions of the gap junction plaques (Figures 7A,B). Similarly, the N54K, R32H, and R184P mutants all localized to distinct locations from Cx43 (Figure 7A). However, the S183F mutant not only colocalized with Cx43 into the same gap junctions, but high magnification enface images revealed that the Cx26 S183F mutant and Cx43 were evenly distributed throughout the gap junction plaque suggesting that they intermixed indicative of a gain-of-function channel characteristic (Figure 7B).

### Cx43 and GJIC Are Not Necessary for the Partial Differentiation of HEI-OC1 Cells

**FIGURE 8:**
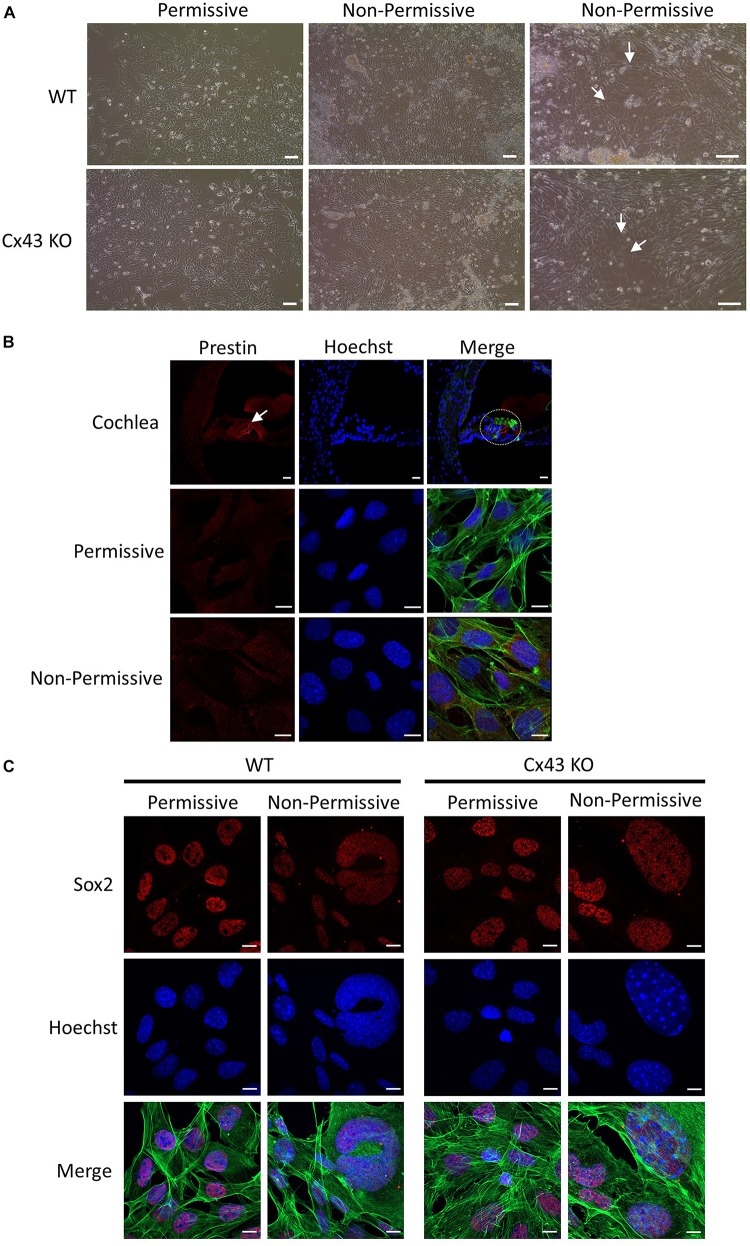
HEI-OC1 cells enlarged but failed to exhibit characteristics of hair cell-like cells when induced to differentiate. **(A)** Light microscope images of WT and Cx43 KO HEI-OC1 cells grown in permissive and non-permissive conditions for 10 days. Arrows denote large cells that appear to have undergone some level of differentiation. **(B)** The motor protein prestin (red) was successfully detected in outer hair cells of a postnatal mouse cochlea cross section (dashed oval) but was not detected in WT HEI-OC1 cells grown in permissive or non-permissive conditions. **(C)** The progenitor cell marker Sox2 (red) expression was detected in HEI-OC1 cells cultured under all conditions. Green = xphalloidin staining, blue = Hoechst stained nuclei. Bars = (A) HEI-OC1 cells = 100 μm, **(B,C)** HEI-OC1 cells = 10 μm, **(B)** cochlea = 20 μm.

**FIGURE 9:**
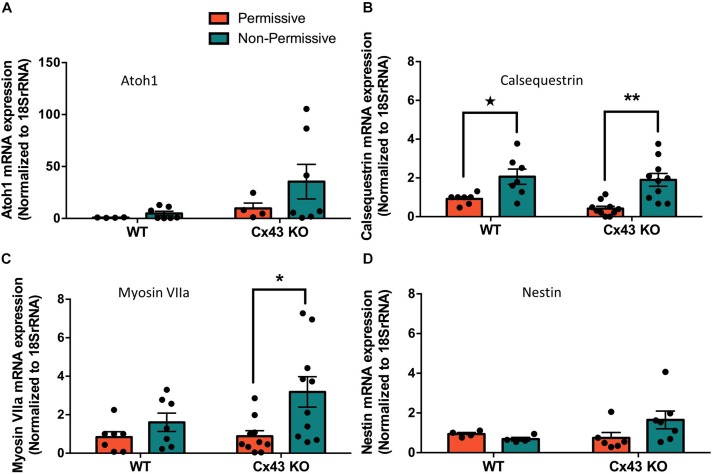
Partial differentiation of HEI-OC1 cells in non-permissive cultures was independent of Cx43 status. **(A)** Real-time qPCR normalized to 18SrRNA revealed that Atoh1 mRNA levels were unchanged when wild type (WT) or Cx43 KO cells were grown for 10 days in non-permissive conditions. **(B)** WT and Cx43 KO cells exhibited increased calsequestrin mRNA levels when cultured in non-permissive conditions, and **(C)** Cx43 KO cells had higher myosin VIIa mRNA levels. **(D)** The progenitor cell marker nestin mRNA expression was unaltered under any conditions tested. Data represent mean ± SEM of four independent experiments comprised of two independent Cx43 KO clones. (*p < 0.05, **p < 0.01 two-way ANOVA and Tukey’s *post hoc*, ★ *p* < 0.05 unpaired *t*-test).

HEI-OC1 cells have previously been shown to differentiate into hair cell-like cells under non-permissive conditions by upregulating hair cell specific molecular markers (Kalinec G. M. et al., 2016). Here we show that after ten days in non-permissive conditions designed to promote cell differentiation, both WT and Cx43 KO cell cultures contained heterogeneous clusters of cells that increased in size (arrows) while others continued to proliferate or undergo apoptosis (Figure 8A). Immunolabeling for the hair cell marker protein, prestin, revealed its detection in outer hair cells of the mouse cochlea, but no prestin was detected in HEI-OC1 cells grown in permissive or non-permissive temperatures (Figure 8B). Consistently, we did not observe the expected decrease in Sox2 labeling, a progenitor cochlear cell marker, in Cx43-negative or WT HEI-OC1 cells which would be expected if these progenitor cells had successfully differentiated into hair cell-like cells. This premise was supported by the fact that the mRNA expression of Atoh1, a transcription factor expressed during the initiation of hair cell development, was not altered under non-permissive conditions in either WT or Cx43 KO cells (Figure 9A). However, we suspected that the HEI-OC1 cells may have partially differentiated but failed to progress to a mature hair cell-like state. In support of this notion, qPCR results provided evidence that mature hair cell markers calsequestrin and myosin VIIa both significantly increased in Cx43 KO cells in non-permissive conditions, whereas calsequestrin also increased in WT cells (Figures 9B,C), suggesting that both the Cx43-rich and null HEI-OC1 cells exhibit some capacity to reprogram genes necessary for cell differentiation. Yet, the mRNA expression of the intermediate filament protein nestin, a stem-cell like marker found in cochlear progenitor cells and downregulated as cochlear development proceeds, was not altered in non-permissive conditions (Figure 9D). Overall, both WT and Cx43 KO cells may have initiated some gene regulation to promote cell differentiation but this was insufficient to drive major cellular changes in the majority of the cells and appeared independent of the presence of Cx43 and overall GJIC.

## Discussion

Understanding the etiology of connexin gene mutations linked to hearing loss remains an essential step for connexin therapeutics and in the emerging therapeutic era of strategic gene editing (Laird and Lampe, 2018). In the past, many non-tissue relevant and often cancerous cell lines have been used to express Cx26 mutants to uncover that *GJB2* gene mutations linked to hearing loss fall into either loss-of-function or gain-of-function mutations (White, 2000; Zhao et al., 2006; Sanchez and Verselis, 2014). However, conclusions from these less than ideal cell culture models must be extrapolated to a cochlear-relevant context and further translated to the *in vivo* setting with the ultimate hope that they inform on the human condition. In the current study we move one step closer to understanding how various *GJB2* gene mutations cause hearing loss by exploring their characteristics in cochlear-relevant cells obtained from the developing organ of Corti. Using this novel strategy we uncovered that autosomal recessive non-syndromic mutants (R32H and R184P) largely traffic and assemble into gap junctions independent of co-expressed cochlear connexins while autosomal dominant syndromic mutants (N54K and S183F) selectively intermixed with Cx26, Cx30, and Cx43. Our findings in HEI-OC1 cells inform on how strategies to up-regulate compensatory connexins may potentially rescue autosomal recessive hearing loss while such strategies may have limited benefit in autosomal dominant disease due to potential connexin isoform intermixing and inactivation.

HEI-OC1 cells that resemble a common progenitor to supporting cells and hair cells were derived from the epithelial region of the organ of Corti in mouse cochlear explants (Kalinec et al., 2003; Kalinec G. et al., 2016; Kalinec G. M. et al., 2016; Kelly et al., 2019). In keeping with most cell lines grown in culture, we observed that Cx43 was abundantly expressed in HEI-OC1 cells. To circumvent the fact that Cx43 is not the predominant connexin isoform found in the mature organ of Corti, we used CRISPR-Cas9 to ablate Cx43 from HEI-OC1 cells and established a Cx43 KO cell platform for the controlled reintroduction of cochlear-relevant connexins and hearing loss-linked mutants. In excess of 135 mutations in the *GJB2* gene encoding Cx26 have been linked to inherited sensorineural hearing loss ranging from moderate to profound severities (Laird et al., 2017) but none of these Cx26 mutants have been investigated in a cochlear-relevant cell line that has retained some capacity for differentiation.

Hearing loss-linked single amino acid substitutions have been identified in each of the five major Cx26 polypeptide domains signifying the importance of each domain (Martinez et al., 2009; Xu and Nicholson, 2013). Here, we chose to examine two mutations linked to syndromic hearing loss (N54K and S183F) (Richard et al., 2004; de Zwart-Storm et al., 2008; Shuja et al., 2016; Press et al., 2017) and two mutations linked to non-syndromic hearing loss (R32H and R184P) (Rabionet et al., 2000; Santos et al., 2005; Mani et al., 2009; Xiao et al., 2011). Our strategy was to use two mutations found within or near each of the 1st and 2nd extracellular loop regions of Cx26. Interestingly, only one of the mutants in each domain caused syndromic disease suggesting that the defect caused by each amino acid substitution can have profoundly different functional outcomes and disease burden.

Both dominant syndromic mutations (N54K and S183F) are found in extracellular loop domains of Cx26 thought to be critical in hydrogen bond-mediated hemichannel docking to form a fully functional gap junction channel (Bruzzone et al., 1996; Maeda et al., 2009). The substitution of asparagine to a lysine (the N54K mutant) results in Cx26 being retained in an intracellular compartment manifesting as the skin pathology Bart-Pumphrey syndrome with accompanying hearing loss (Richard et al., 2004). The intracellular retention of the N54K mutant and its inability to form gap junctions was further supported by our previous studies in HeLa cells and rat epidermal keratinocytes (Press et al., 2017). In the current study, we show that the N54K mutant is indeed retained in an intracellular compartment when expressed alone in GJIC-deficient cochlear-relevant cells. However, when this mutant was co-expressed with Cx30, but not typically with Cx26 or Cx43, it was abundantly found in gap junctions suggesting that Cx30 not only intermixed with the N54K mutant but rescued its delivery to the cell surface and assembly into gap junctions. It is notable that the original patients identified to harbor the N54K mutant exhibited a compensatory upregulation of Cx30 (Richard et al., 2004). Collectively, all lines of evidence point to N54K being a loss-of-function trafficking defective mutant that can be rescued by the co-expression of Cx30, which is typically co-expressed with Cx26 in the organ of Corti and may serve to reduce the severity of hearing loss that might otherwise occur.

The second syndromic mutant (S183F) investigated in HEI-OC1 cells exhibited characteristics of being able to form gap junctions. This serine to phenylalanine substitution linked to palmoplantar keratoderma and hearing loss (de Zwart-Storm et al., 2008) was previously shown to form non-functional gap junctions in HeLa cells (Shuja et al., 2016; Press et al., 2017) suggesting that the hydrogen bonding generated by the 2nd extracellular loop is sufficiently retained to ensure hemichannel docking in the assembly of functionally dead gap junctions. In keeping with Shuja et al. (2016) who provided evidence that the S183F mutant may have exhibited the gain-of-function characteristic of being able to intermix with Cx43 into heteromeric and heterotypic channels (Shuja et al., 2016), we also found that the S183F mutant fully intermixed with Cx43 within gap junctions formed in cochlear-relevant cells. These findings are unexpected as normally Cx26 and Cx43 are unable to co-oligomerize and remained segregated to different subdomains of the gap junctions (Falk, 2000), although other Cx26 mutants linked to skin diseases have been shown to intermix with Cx43 (Garcia et al., 2015). Furthermore, the connexin motifs that have been reported to govern oligomerization are believed to be localized between the amino terminal and 3rd transmembrane domain (Martinez et al., 2011) which are distant sites from the location of the S183F mutation. Collectively, these studies point to a new role for the 2nd extracellular loop in governing connexin oligomerization properties. Interestingly, the S183F mutant also retained the properties of being able to reside in both Cx26 and Cx30 gap junctions suggesting that its gain-of-function characteristics of being able to intermix with Cx43 did not come with a concomitant loss of the ability to intermix with these critical cochlear connexins.

Intriguingly, even though Arg184 resides next to Ser183 within the second extracellular loop, homozygous *GJB2* allelic mutations resulting in an arginine to proline (R184P) change only causes hearing loss while heterozygous carriers of this mutation are unaffected (Mani et al., 2009). This arginine residue is thought to be important for inter-protomer interactions between the second extracellular loop and the adjacent connexon (Maeda et al., 2009). The R184P mutant has been reported in reference cells to exhibit some trafficking deficits with an inability to form gap junctions (Bruzzone et al., 2003; Mani et al., 2009; Xiao et al., 2011) and may be prematurely degraded (Thonnissen et al., 2002; Mani et al., 2009). When expressed in cochlear-relevant cells, we found that gap junctions were rarely assembled and this mutant frequently localized to early endosomes suggesting it may have reached the cell surface before being retrieved for imminent degradation in lysosomes.

The R32H mutation resides within the 1st transmembrane domain near the 1st extracellular domain; a polypeptide motif that likely has multiple roles in the formation of intra-protomer interactions, connexin folding, oligomerization, and channel pore formation (Maeda et al., 2009). Little is known about the fate of the autosomal recessive mutation that encodes the R32H mutant (Mustapha et al., 2001), although one study showed that it localized to the endoplasmic reticulum when expressed in HeLa cells (Xiao et al., 2011). This did not seem to be the case when this R32H mutant was expressed in HEI-OC1 cells as it partially localized to early endosomes with no clear evidence that it was trapped in the endoplasmic reticulum. Thus, this mutant appears to be following a similar fate as the R184P mutant and maybe destined for premature degradation. This finding highlights the importance of examining hearing loss linked mutants in a cochlear-relevant system as their expression within different tissue types may lead to distinctly different outcomes. Our results support the notion that non-syndromic mutants do not acquire gain-of-function properties but cause hearing loss via their loss-of-function and premature targeting to the degradation pathway.

Finally, we wanted to determine if the state of GJIC altered the ability of HEI-OC1 cells to differentiate toward supporting cell and/or hair cell fates. Since Cx43 is endogenously expressed in these cochlear-relevant cells and Cx43 channels typically allows for permissive small molecule exchanges that exceeds the scope of gap junction exchange that can occur through Cx26 channels (Harris, 2008; Lopez et al., 2014), we used these cells as a surrogate for GJIC that occurs *in vivo* and assessed cell differentiation before and after Cx43 ablation. We also knew that GJIC is critically important in the organ of Corti as hair cells are deformed and the tunnel of Corti formed by supporting cells is absent in conditional Cx26 null mouse models (Inoshita et al., 2008; Chang et al., 2015; Zhu et al., 2015; Chen et al., 2018a). Further, transgenic mice expressing the dominant Cx26 mutant R75W displayed delayed apoptosis during cochlear development and the organ of Corti was malformed (Inoshita et al., 2014). In our studies, we found HEI-OC1 cells were not able to fully differentiate into hair cell-like cells expressing protein markers and genes of *in vivo* hair cells, including prestin, even though prestin has previously been reported to be expressed in differentiated HEI-OC1 cells (Kalinec G. M. et al., 2016; Park et al., 2016). We suspect that the HEI-OC1 cells we used are somewhat heterogeneous and may have lost their full potential to differentiate into hair cell-like cells in non-permissive conditions since their original isolation nearly 20 years ago (Kalinec et al., 2003). Nevertheless, we found that there were pockets of cells within the cultures that appeared to differentiate amongst unchanged cells supporting the notion that the cultures had mixed cell phenotypes. Others have noted this heterogeneity amongst different batches of HEI-OC1 cells (Cederroth, 2012). In our studies, we did find that the ablation of Cx43 drove a higher expression of myosin V11a and calsequestrin suggesting that Cx43 mediated GJIC may be acting as a negative regulator of some genes important in cell differentiation. Calsequestrin was also elevated in wild type cells and Sox2 was abundantly found in both Cx43 positive and negative cultures. These findings are all consistent with a small subpopulation of HEI-OC1 cells retaining the capacity to differentiate with the majority of the cells remaining in a progenitor state. Nevertheless, these cells remain the best cochlear-relevant cell line to interrogate the functional status of hearing loss-linked Cx26 mutants.

In summary, sensorineural hearing loss linked to *GJB2* gene mutations is one of the most common inherited conditions found worldwide affecting as many as 1/2000 live births (Chan and Chang, 2014). Our understanding of how these mutations induce hearing loss is still emerging and must be fully understood to establish a platform for tactical drug design and rational treatment strategies. Here, we used a cochlear-relevant cell line to further investigate two syndromic and two non-syndromic Cx26 mutants that are found within or near the extracellular loop regions of the connexin polypeptide. Collectively, we found that each of the syndromic mutations exhibited unique gain-of-function properties while the two non-syndromic mutants exhibited common loss-of-function characteristics.

## Data Availability Statement

All datasets generated for this study are included in the article/supplementary material.

## Ethics Statement

The animal study was reviewed and approved by the Animal Care Committee at the University of Western Ontario.

## Author Contributions

RB performed the bulk of the experiments, prepared the figures and drafted the manuscript. JA trained RB, cultured cells, prepared the mouse material and proof-read the manuscript. BA obtained the cells used, provided advice and proof-read the manuscript. JE assisted in engineering the Cx43-ablated cells and proof-read the manuscript. QS oversaw the generation of the Cx26 mutants and proof-read the manuscript. DL supervised the project, obtained funding and refined the manuscript for submission.

## Conflict of Interest

The authors declare that the research was conducted in the absence of any commercial or financial relationships that could be construed as a potential conflict of interest.
